# Effectiveness of Transitional Care in Inflammatory Bowel Disease; Development, Validation, and Initial Outcomes of a Transition Success Score

**DOI:** 10.1093/ecco-jcc/jjae166

**Published:** 2024-11-01

**Authors:** Martha A C van Gaalen, Merel van Pieterson, Petra Waaijenberg, Angelika Kindermann, Victorien M Wolters, Alie Dijkstra, Herbert van Wering, Margreet Wessels, Lissy de Ridder, Dimitris Rizopoulos, C Lauranne A A P Derikx, Johanna C Escher, the Kids with Crohn’s, Colitis (KiCC) Working Group for Collaborative Paediatric IBD Research in the Netherlands, the Dutch Initiative on Crohn and Colitis (ICC) and Dutch Nurses Network Inflammatory Bowel Disease (NIBD), Patrick F van Rheenen, Sarah T A Teklenburg, Fiona D M van Schaik, Janneke C van der Woude, Jildou Hoekstra, Marleen de Leest, Carla Bakker, Elvira M E Besuijen-Laterveer, Esther Adriaanse, Jolien Wisse, Marloes Heida, Pamela Hurkmans, Nynke Boontje, Tessa Z Toonen, Wendy Heida

**Affiliations:** Department of Paediatric Gastroenterology, Erasmus MC-Sophia Children’s Hospital, Rotterdam, The Netherlands; Department of Paediatric Gastroenterology, Erasmus MC-Sophia Children’s Hospital, Rotterdam, The Netherlands; Department of Gastroenterology, Amsterdam UMC, Amsterdam, The Netherlands; Department of Paediatric Gastroenterology, Amsterdam UMC – Emma Children’s Hospital, Amsterdam, The Netherlands; Department of Paediatric Gastroenterology, UMC Utrecht – Wilhelmina Children’s Hospital, Utrecht, The Netherlands; Department of Paediatric Gastroenterology, UMC Groningen – Beatrix Children’s Hospital, Groningen, The Netherlands; Department of Paediatrics, Amphia Hospital, Breda, The Netherlands; Department of Paediatrics, Rijnstate Hospital, Arnhem, The Netherlands; Department of Paediatric Gastroenterology, Erasmus MC-Sophia Children’s Hospital, Rotterdam, The Netherlands; Department of Biostatistics, Erasmus MC, Rotterdam, The Netherlands; Department of Gastroenterology, Erasmus MC, Rotterdam, The Netherlands; Department of Paediatric Gastroenterology, Erasmus MC-Sophia Children’s Hospital, Rotterdam, The Netherlands

**Keywords:** IBD, inflammatory bowel disease, young adults, transition

## Abstract

**Background and Aims:**

The effectiveness of transition programs from pediatric to adult healthcare in adolescents with inflammatory bowel disease (IBD) is not clear, as prospective studies using validated outcome measures for transition are lacking. This study aimed to develop and validate a quantitative Transition Success Score (TSS), and to apply it in a multicenter setting to assess the effectiveness of transitional care.

**Methods:**

The Top 10 outcome items related to a successful transition, identified through an international Delphi study with IBD stakeholders, were integrated into a generic questionnaire, the TSS. In a prospective, multicenter study, the TSS was scored by adult healthcare providers, young adult patients, and caregivers, 9-15 months after transfer of care.

**Results:**

In 7 Dutch hospitals, 160 patients completed the TSS. The mean score was 25 (range 17-27), 25.6% of patients achieving maximum score. Hypothesis testing for construct validity revealed significant associations with characteristics related to transitional care, such as knowledge, independence, and quality of life (*p* < 0.005). Structural validation indicated the score was most effective at discerning lower levels of transition success. Internal consistency was acceptable (0.64). High disease burden, exacerbation during or after transfer, and certain personality profiles were associated with lower scores.

**Conclusions:**

The TSS serves as a quantitative tool to evaluate the effectiveness of transitional care interventions and to identify IBD patients at risk of encountering challenges during the transition to adult healthcare.

## Graphical Abstract

**Graphical Abstract:**
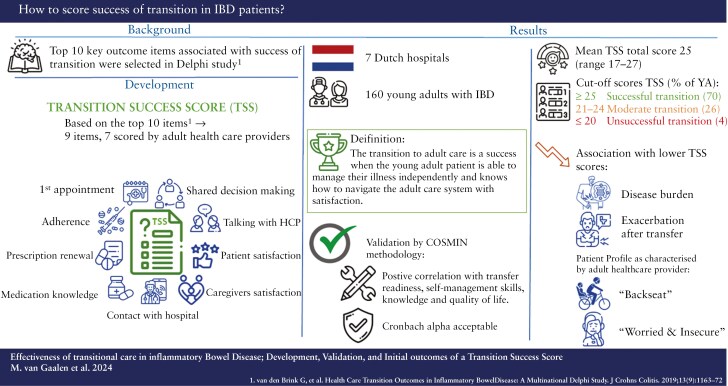

## 1. Introduction

Successful transition from pediatric to adult healthcare is crucial for the health and well-being of young adults (YAs, aged 18-25 years) with chronic conditions such as inflammatory bowel disease (IBD), including Crohn’s disease and ulcerative colitis.^1,2^ However, ensuring structured and high-quality transitional care for all patients to achieve a successful transition poses challenges. It requires a strong commitment of healthcare providers to support YA patients in navigating the changes they will encounter in adult healthcare while considering their physical, psychological, and social health status.^1,3,4^ Additionally, caregivers must allow their adolescent children to develop independence gradually. Moreover, the empowerment of YA patients with disease knowledge, autonomy, self-efficacy, and self-management skills is crucial.^2,5–7^ These aspects can be particularly challenging as they coincide with a developmental period filled with changes in many areas, such as school, work, housing, and relationships.

The implementation of a structured transition program in IBD has been reported to improve the YA’s self-management skills, disease knowledge, and quality of life (QoL).^1,2,8–10^ There is however no evidence favoring 1 transitional care model over others due to a lack of prospective studies using a clear definition of transition success.^11,12^ Studies evaluating transition success often rely on non-validated, or qualitative tools based on patients’ experience or satisfaction.^11–15^

Previously, our research group conducted a Delphi study identifying key outcomes of transition success for IBD patients.^16^Table 1 displays the Top 10 items determining transition success (of increasing importance, so number 10 as most important item), selected by an international panel of adolescent and YA IBD patients, pediatric and adult gastroenterologists, and specialized nurses. The most crucial factor was “*demonstration of decision-making skills in relation to IBD*” as well as other self-management skills that were prioritized over IBD-specific factors such as “*frequency of exacerbations*” and “*surgery*.” In the absence of a clear definition, the following definition of transition success was used for this study, based on the findings of this top 10 list: The transition to adult care is a success when the YA patient is able to manage their illness independently and knows how to navigate the adult care system with satisfaction.^16,17^

**Table 1:** Top 10 items associated with transition success (16).

Attends first adult healthcare appointment within the first 3-6 months after transfer
Contacts IBD service independently
Attends first adult healthcare appointment as planned at moment of transfer
Fills own prescriptions on time
(Health related) Quality of Life 1 year after transfer
Ability to recall dose/frequency of medication
Medication adherence
Patient satisfaction with transition process
Independent communication with treating physician/nurse
Shows ability to make decisions regarding IBD

Abbreviation: IBD, inflammatory bowel disease.

Our Delphi study was the starting point of the development of an outcome measure to assess transitional care effectiveness. The present multicenter study in YAs with IBD aimed to (1) create and validate a Transition Success Score (TSS) based on the Top 10 list from our Delphi study and (2) prospectively use this TSS score to evaluate the outcome of transitional care in a multicenter setting.

## 2. Materials and Methods

The COSMIN methodology^18^ guided the development and validation of a Patient Report Outcome Measurement. An international consensus procedure was utilized to create the TSS, which underwent initial testing in a pilot study. Subsequently, a multicenter prospective validation study was conducted using the finalized TSS.

The Research Ethics Review Board of the Erasmus University Medical Center approved this study (MEC-2017-459). Informed consent was obtained from all patients before recruitment into the study.

### 2.1. Development of the TSS

All 64 healthcare experts who participated in our previous Delphi study^16^ were contacted to be involved in the development of the TSS, based on the outcomes of our previous Delphi study (Table 1). Of 64 international healthcare experts, 60 confirmed their availability (see Supplementary Table 1 for demographic characteristics).

The initial version of the English language TSS was developed by our research team (MvG, JCE). For each item on the Top 10 item list, we formulated a single question with 3 answer options. Four rounds of the Delphi process were conducted between October 2019 and July 2020 using the online survey platform SurveyMonkey. These rounds aimed to collect feedback (agreement or disagreement) from participating experts regarding the formulated questions and answer options. Each round allowed a 4-week time frame for participants to submit their responses, with reminders sent every week to non-respondents. The survey link was closed after 3 reminders were sent or when the response rate reached 80%.

**Figure 1:**
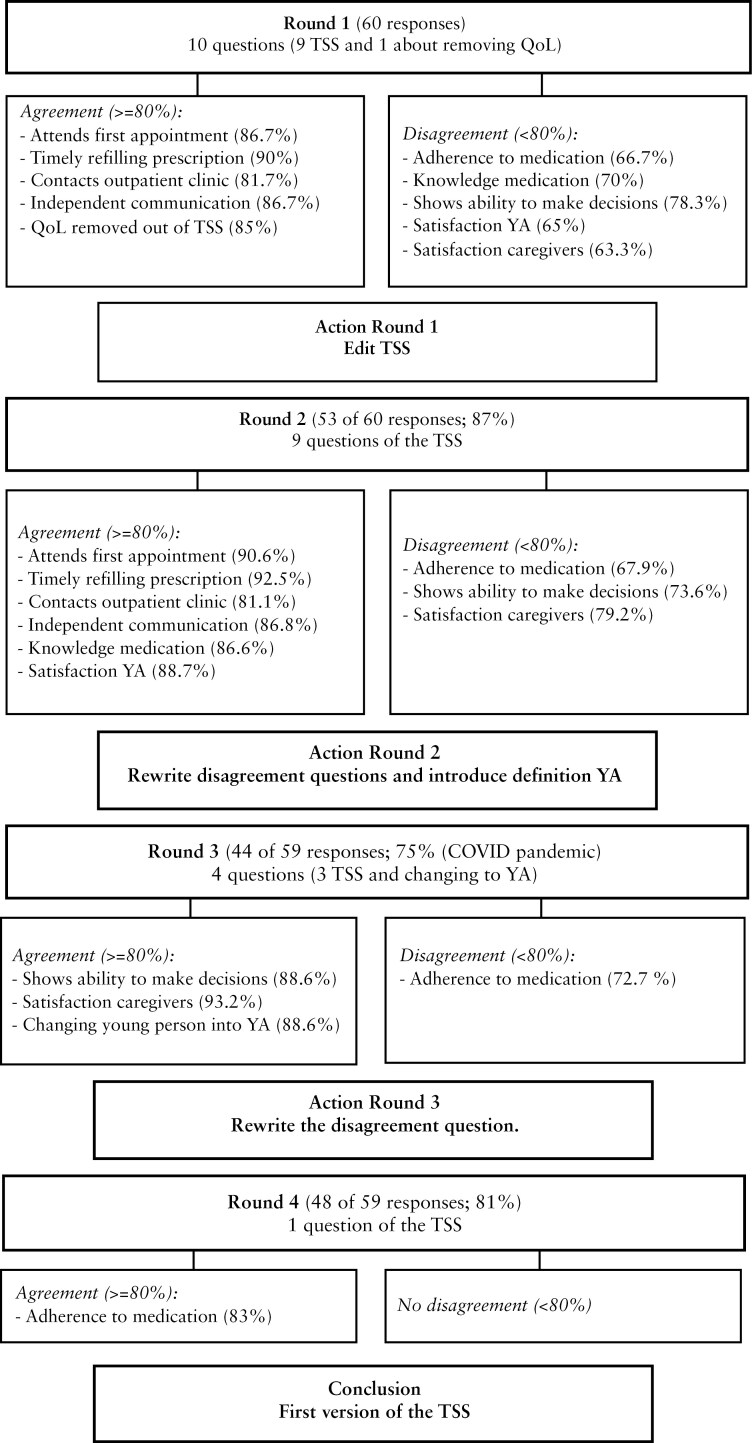
Overview Delphi study; consensus development. The listed items are the Top 10 items associated with a successful transition. Abbreviations: TSS, Transition Success Score; QoL, quality of life; YA, young adult.

Figure 1 shows that consensus (agreement above 80%) was reached on 9 items of the TSS after 4 rounds. During the first round, the experts discussed the complexity of measuring and establishing a relationship between QoL and transition success. It was then decided not to include QoL in the TSS, but to use it as a validation measure, as previous studies have shown that good quality transitional care can lead to better QoL.^15,19,20^

#### 2.1.1. The first version of the TSS

Following the 4 Delphi rounds, the TSS included 7 items regarding patient’s disease management (assessed by healthcare providers) and 2 items related to patient satisfaction (assessed by both the patient and caregiver). The maximum cumulative score of the TSS is 27, with higher scores indicating a more successful transition.

#### 2.1.2. Pilot study with the first version of the TSS

From August to October 2020, we performed a pilot study to assess the reliability and ease of completing the first version of the TSS among 44 IBD patients (age range 18.7-20.8 years) who had 9-15 months earlier transitioned to adult care in Erasmus MC, Rotterdam, The Netherlands. YA also completed the Rotterdam Transition Test (RTT) to assess disease knowledge,^6^ the Transition Readiness Assessment Questionnaire (TRAQ-NL) to assess self-management skills,^5^ and the Inflammatory Bowel Disease Questionnaire (IBD-Q) to assess QoL.^21^ Efficient completion of the TSS within 5 minutes was demonstrated, and its reliability was deemed satisfactory (Cronbach’s alpha = 0.78). Since not all questions achieved a Cohen’s kappa score exceeding 0.61 (ranging from 0.44 to 1.00), modifications were made to enhance the differentiation between response options and provide additional clarification where necessary (Table 2; final version of the TSS).

**Table 2:** Final version of the Transition Success Score with the total scores of the YA.

Question (by who)	Answer options (how to score) {awarded points}	Score of all YA (*n* = 160) *n* (%)
Q1 (by professional) Has the YA (adolescent/young adult) attended their first appointment at the adult GI department?	A1 (chart review)	
	Yes, YA attended at the first appointment at the adult GI department (*also applies if the YA has rescheduled, in short term, the appointment for legitimate reasons*). {3}	142 (89)
	No, the YA did not attend the first appointment, but the YA did attend a new appointment within 6 months after the last visit in pediatric care. {2}	14 (9)
	No, YA did not attend any visit at the adult GI department within 6 months. {1}	4 (2)
Q2 (by professional) Has the YA adhered correctly to their medical treatment (oral, rectal, infusion, injections) since last visit?	A2 (estimate of the professional, checking during visit)	
	Good adherence; took medication always or most of the time (>*80% of doses*). {3}	143 (90)
	Partial adherence: did not take medication at all times *or* did not take all types of medication (eg, *good adherence to oral, but not rectal medicatio*n). {2}	12 (7)
	Significant non-adherence: stopped all or most medication *or* did not show up for infusions. {1}	5 (3)
Q3 (by professional) Does the YA show that he/she can organize a renewal of their prescription(s) by themselves? Or in case of infusion medication: Has the YA planned their infusions independently?	A3 (chart review on request renewal of prescription OR check by YA if he/she know how to arrange)	
	Yes, YA shows that he/she can organize a renewal of their prescription/infusion appointment *by themselves*.{3}	133 (83)
	YA organized a renewal of their prescription/infusion appointment *together with caregiver(s)*. {2}	21 (13)
	No, renewal of their prescription/infusion appointment always organized by *the caregiver(s)*. {1}	6 (4)
Q4 (by professional) Has the YA been able to recall name/dose/frequency of his/her medication?	A4 (check during visit)	
	Yes, YA was able to recall name *and* dose *and* frequency correctly (*in case of infusion therapy: able to recall name and dosing interval only*). {3}	147 (92)
	No, YA could only recall name *or* dose/frequency correctly. {2}	12 (7)
	No, YA was not able to give correct answers regarding any medication {1}	1 (1)
Q5 (by professional) When the YA has a question relating to their disease, does they contact the hospital and health care professionals themselves?	A5 (chart review on who contacts OR check by/assess during visit)	
	Yes, the YA will/is able to contact the hospital by themselves. {3}	131 (82)
	Sometimes caregivers make contact *or* YA needs help from caregivers to contact the hospital. {2} No, the YA is unable to contact the hospital *or* caregiver(s) have always contacted the hospital and health care professionals. {1}	25 (16)
		4 (2)
Q6 (by professional) Does the YA talk independently with the treating physician/nurse?	A6 (check during visit)	
	Yes. Caregivers are allowed to be present during the appointment but ask almost no questions (*comparable to a partner who is present during an appointment with an adult patient*). {3}	145 (91)
	YA needs help from caregivers to ask/answer certain questions *or* to be reminded to ask something. {2}	13 (8)
	No, most (>50%) of the communication went through the caregiver(s). {1}	2 (1)
Q7 (by professional) Do you feel that the YA is involved in shared decision making, and that the YA can discuss independently with the health care provider about treatment, tests and other disease-related issues?	A7 (assessment of the professional, during visit)	
	Yes, the YA is independently involved in shared decision making and contributes in the care process. If necessary, the YA consults with a caregiver on an equivalent level (*comparable to a partner who is present during an appointment with an adult patient*). {3}	131 (82)
	Yes, but the YA needs help/guidance from the caregiver(s) in shared decision making (*for example, in the final decision making*) *or* caregivers lead a major part of the conversation. {2}	28 (17)
	No, there is no shared decision making with the YA (*no share decision making at all or only by the caregiver(s)*) {1}	1 (1)
Q8 (by YA) How satisfied are you with the transition process? Give a score (1 = completely unsatisfied and 10 = completely satisfied)	A8 (ask the YA)	
	Satisfied (score 8-10) {3}	99 (62)
	Neutral (score 5-7) {2}	57 (36)
	Unsatisfied (score 1-4) {1}	4 (2)
Q9 (by caregiver) How satisfied are you, as a caregiver, with the transition process? Give a score (1 = completely unsatisfied and 10 = completely satisfied)	A9 (ask the caregiver)	
	Satisfied (score 8-10) {3}	85 (53)
	Neutral (score 5-7) {2}	71 (44)
	Unsatisfied (score 1-4) {1}	4 (3)

Abbreviations: A, answer option; TSS, Transition Success Score; Q, question; YA, young adult.

### 2.2. Validation of the TSS

Validation was carried out using the COSMIN method, which involves construct validation, structural validation, and measurement of reliability.

#### 2.2.1. Patients

Between May 2021 and April 2023, 7 Dutch hospitals (4 academic and 3 non-academic hospitals) enrolled YA with IBD who had transitioned to adult healthcare 9-15 months before. This time frame was chosen to ensure that YAs were used to the responsibilities and routines of adult care and could still recall the transfer experience. Following the COSMIN methodology, our goal was to collect 150 completed TSS, which is deemed sufficient for validation purposes.^18^ All eligible YAs were invited to participate, and in cases of refusal, reasons for declining were documented. Although transition programs varied between the hospitals, most had a transition protocol or clinic to focus on transitional care within the local setting. Following standard practice in the Netherlands, patients are usually transferred to adult care around 18 years of age.

#### 2.2.2. Data collection

The receiving adult healthcare providers were responsible for reporting on 7 of the 9 items of the TSS during an outpatient visit. Concurrently, YAs and their caregivers were instructed to digitally respond to the 2 satisfaction-related questions, translated into Dutch. YA also completed validated questionnaires related to transition; the RTT (knowledge),^6^ TRAQ-NL (self-management),^5^ and the IBD-Q (QoL).^21^ Transfer readiness assessments were collected from YA, pediatric, and adult healthcare providers using a 100-point visual analog scale (VAS). In addition, YA self-assessed their level of independence using a VAS. All questionnaires were requested to be completed within a 4-week period, encompassing 2 weeks before a routine outpatient visit to 2 weeks after. The questionnaires were distributed using Lime Survey version 3.1.1 (LimeSurvey GmbH, Hamburg, Germany).

To evaluate the construct validity of the TSS, we examined the correlation between the TSS total score and self-management skills and transition readiness characteristics (Table 3). Since no major changes were made to the TSS after the pilot phase, the final validation analysis included the TSS outcomes as along with the results of the TRAQ, RTT, and QoL assessments from the pilot study.

**Table 3:** Construct validation of the TSS with hypothesis testing.

Association of total TSS scores of patients with IBD with specific variables:	Measurement	Pearson correlation	*p*-value
TSS should correlate positively with higher VAS self-management scored by YA	VAS self-management YA	0.348	**<0.001**
TSS should correlate positively with higher VAS transfer readiness scored by YA	VAS transfer readiness YA	0.317	**<0.001**
TSS should correlate positively with higher VAS transfer readiness scored by pediatric health care providers	VAS transfer readiness pediatric HCP	0.279	**<0.001**
TSS should correlate positively with higher VAS transfer readiness scored by adult health care providers	VAS transfer readiness adult HCP	0.584	**<0.001**
TSS should correlate positively with higher level of self-management	TRAQ-NL (5)	0.444	**<0.001**
TSS should correlate positively with higher level of disease knowledge	RTT (6)	0.308	**<0.001**
TSS should correlate positively with higher level of quality of life	IBDQ (21)	0.242	**0.004**

Abbreviations: HCP, healthcare providers; IBD, inflammatory bowel disease; IBDQ, IBD questionnaire; QoL, quality of life; RTT, Rotterdam Transition Test; TRAQ, Transition Readiness Assessment Questionnaire; TSS, Transition Success Score; VAS, visual analog scores; YA, young adult.

Bold values represent significant difference; *p* = <0.005.

### 2.3. Predictors of successful transition

To identify predictive factors regarding the success of transition, demographic information, disease behavior, and disease activity assessment at the time of transfer and at the time of completion of the TSS were collected through chart review and a supplementary questionnaire for YA on education level, family composition, patients’ and caregivers’ country of birth. YA conducted self-assessments on disease acceptance using a 4-point Likert scale response to the question, “*I can accept that I will have this disease for the rest of my life*.” Additionally, perceived disease burden was self-assessed on a 4-point Likert scale in response to the question, “*How much do you currently suffer from your illness?*” Identification of IBD exacerbation between transfer and completion of the TSS was based on the initiation of or changes in medication due to increased symptoms or the presence of endoscopic disease activity. Disease activity was scored according to the Physician Global Assessment (PGA), which categorizes disease activity as normal, mild, moderate, or severe.

A previous study using Q methodology categorized a group of chronically ill YA into 4 personality and behavioral profiles.^22^ The “*Conscious & Compliant*” profile denoted individuals with a high level of involvement in disease knowledge and management. The “*Backseat Patient*” profile characterized YA who were less mature and relied more on their caregivers. Patients expressing a strong desire to be transparent about their disease, not concealing it but embracing it in their daily lives, were classified as “*Self-confident & Autonomous*.” The “*Worried & Insecure*” profile encapsulated patients primarily concerned about their disease. In the present study, both pediatric and adult healthcare providers utilized these profiles to determine the patient type for each YA within the context of transition.

### 2.4. Statistical analysis

Statistical analyses were performed conducted SPSS for Windows, version 28.0.1.0 (IBM SPSS Statistics for Windows, Armonk, NY, USA). Significance was determined at a *p*-value <0.05. Only fully completed TSS questionnaires were included in the analysis. Incompletely answered supplementary questionnaires were excluded from the analysis, leading to fewer participants for the corresponding analysis.

#### 2.4.1. Validation phase

To compare the independent variables of the hypothetical constructs in Table 3, *t*-tests were used for dichotomous variables and Pearson’s correlation coefficients for continuous variables. Structural validation was conducted using Rasch analysis, a method that assesses question characteristics, identifies the need for question modification, and evaluated the questions’ ability to distinguish between different levels of success of transition, which is the latent variable. This latent variable is normally distributed with a mean of 0 and a variance of 1. A value of 0 represents the mean transition success, and the range of all values is from −4 to 4.^23^ The reliability of the entire TSS was evaluated using Cronbach’s alpha.

#### 2.4.2. Predictors of successful transition

Associations between various predictor variables and total TSS scores were assessed using univariable and multivariable analyses. Due to low response frequencies, the estimation of the model’s coefficients was not stable, the questions concerning disease activity (PGA), disease burden, and acceptance of disease, all rated on a 4-point scale, were merged into 2 categories (present vs absent). An analysis of variance was carried out to explore the relationship between education level and TSS score as well as family composition and TSS score. The dependent variable was TSS, while the categorical predictor was either education level or number of siblings. For comparing Q-patient profiles, the Fisher–Freeman–Halton exact test was used due to the nominal nature and low frequencies in some profiles.

#### 2.4.3. Cutoff scores

To enhance the practicality of the TSS in routine clinical care, cutoff scores need to be determined. In the absence of a gold standard, these scores were determined by an expert panel. Most importantly, the cutoff scores should enable identification of the YAs who had a problematic transition and transfer of care, and who are in most need of individualized transitional care. In light of the aforementioned premises and the previously described definition of transition success, along with the questions posed by the TSS, the following score distribution was established: Transition was successful with total TSS scores ≥25, moderately successful with scores of 21-24, and scores ≤20 were indicative for unsuccessful transition.

## 3. Results

### 3.1. YA patients

**Figure 2:**
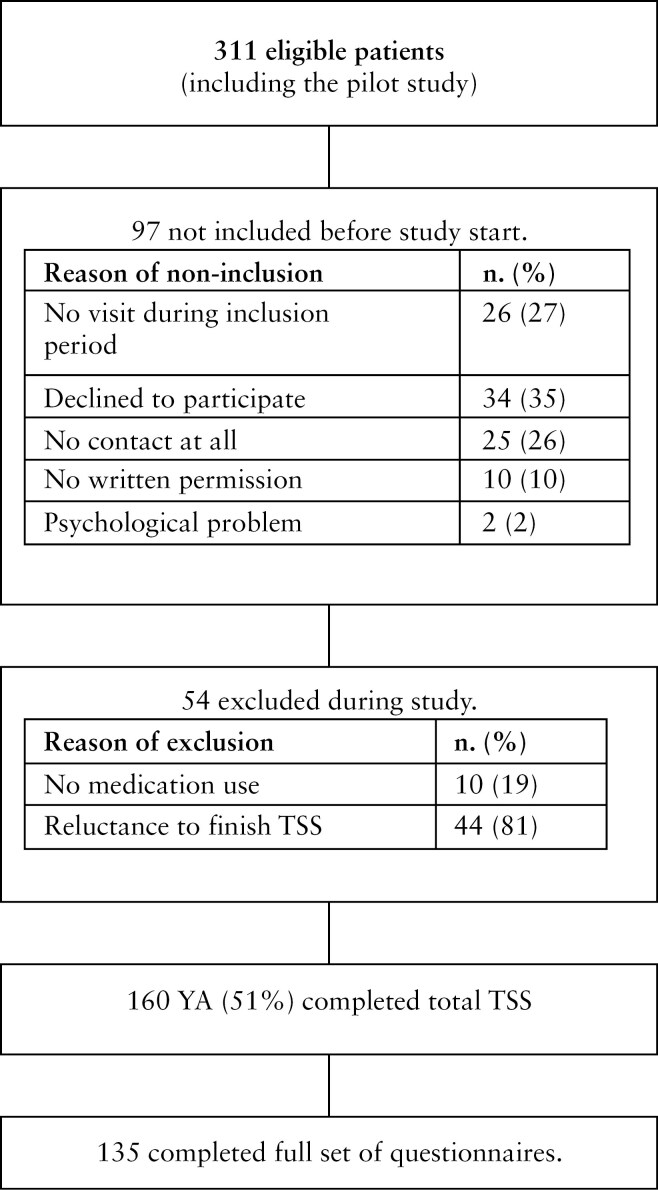
Inclusion procedure TSS validation study. Abbreviations: TSS, Transition Success Score; YA, young adult.

One hundred and sixty of the 311 IBD-eligible patients (meeting the inclusion criteria) completed the TSS (response percentage 51%), of whom 49% were male and 56% had Crohn’s disease. The mean age at diagnosis was 14 years (IQR 3.56). One hundred and thirty-five participants completed the full set of questionnaires (Figure 2). Table 4 shows the demographics of participating patients as well as patients who were not included. Reasons for not willing to participate are reported in Figure 2. A significantly higher proportion of non-included patients were male (*p* = 0.02), patients with disease in clinical remission (*p* = 0.04), and had a significantly lower transfer readiness score as reported by their pediatrician (*p* = 0.005). Additionally, non-included patients were significantly (*p* = 0.03) more often identified as “*Self-confident & Autonomous*.”

**Table 4:** Demographics of included (*n* = 160) and excluded patients (*n* = 151).

	*N* (%) or mean Inclusion patients	*N* (%) or mean (SD) Exclusion patients	*p*-value
Sex			
Male, %	78 (49)	94 (61)	**0.02**
IBD diagnosis			ns
Crohn’s disease	89 (55.6)	87 (56.6)	
Ulcerative colitis	66 (41.3)	61 (39.6)	
IBD-U	5 (3.1)	6 (3.9)	
Disease activity at moment of transfer			
Remission	96 (60)	107 (71.3)	**0.04**
Q-Profile YA (as reported by pediatric healthcare provider)			Versus profile “*Conscious & Compliant*”
“Conscious & Compliant”	90 (68.2)	68 (49.6)	
“Backseat patient”	14 (10.6)	25 (18.2)	ns
“Self-confident & Autonomous”	15 (11.4)	30 (21.9)	**0.03**
“Worried & Insecure”	13 (9.8)	14 (10.2)	ns
VAS transfer readiness (as reported by pediatric healthcare provider)	Mean 82.95 (12.59) IQR 15	Mean 78,2 (13.3) IQR 20	**0.005**

Abbreviations: IBD, inflammatory bowel disease; IQR, interquartile range; ns, not significant; *r*, Pearson correlation; VAS, visual analog scores; YA, young adult.

Bold values represent significant difference; *p* = <0.05.

After transition and transfer, adult healthcare providers rated the YAs more often as “*self-confident and autonomous*” (*n* = 106, 80.3%) compared to the pediatricians before the transition (*n* = 90, 68.2%).

### 3.2. Validation of TSS

#### 3.2.1. Structural validation of the TSS

**Figure 3:**
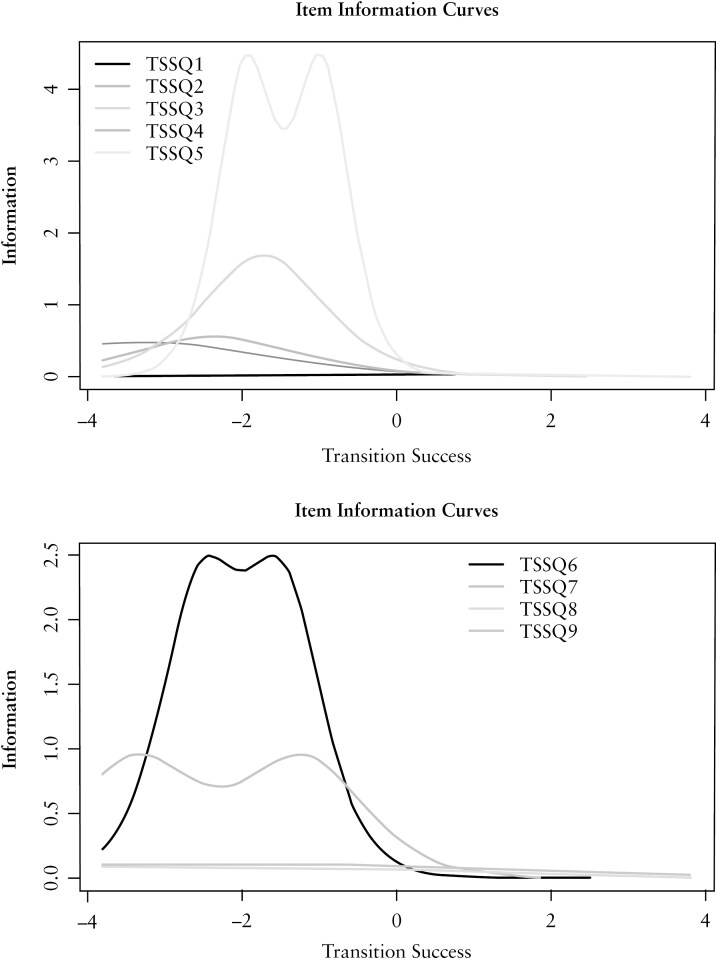
Item information curves. Test Information Function showing per question how much information it provides (*x*-axis) at the transition success level (*y*-axis). Abbreviations: Q, question; TSS: Transition Success Score. −4 totally failed transition to 4 perfectly successful transition.

Figure 3 depicts the results of the Rasch model per question, which ranges from −4 to 4. The figure illustrates that the TSS is most effective in discerning between low (−4) and moderate (0) levels of transition success. This implies that the TSS can best identify patients who did not have a successful transition. Questions with the lowest discriminative value were those on “*attended first appointment*” (TSSQ1) and “*satisfaction scored by patient and the caregiver*” (TSSQ8 and TSSQ9).

#### 3.2.2. Construct validity with hypothesis testing

All questionnaires pertaining to transition readiness and self-management skills, including TRAQ-NL, RTT, and QoL, correlated significantly, positively with the TSS (Table 3). Transfer readiness as scored by the adult healthcare providers using VAS had the highest correlation, albeit still relatively weak (*r* 0.584; *p* < 0.001). The most robust relationship was observed between the TSS and a questionnaire was self-management skills assessed by TRAQ-NL (*r* 0.444; *p* < 0.001).

#### 3.2.3. Reliability of the TSS

Cronbach’s alpha for internal consistency of the TSS was 0.64, demonstrating acceptable reliability of the total TSS.

### 3.3. Outcome and predictors of the TSS

The mean TSS score was 25 (score range 17-27), with a quarter of YA patients achieving the maximum score. Table 2 indicates that 92% of the participants achieved the maximum score on question 4 about recalling medication. Of the caregivers, 53% scored a maximum VAS score (of 10) for their satisfaction concerning the transition process. Caregivers and YAs were satisfied with the transition care program (mean 7.4; SD 1.5 vs 7.8; SD 1.5).

#### 3.3.1. Cutoff scores

The transition was successful (total TSS score ≥25) in 112 patients (70%). Transition success was moderate in 42 patients (26.2%) who had a total TSS score between 21 and 24. Transition was unsuccessful (TSS ≤20) in 6 patients (3.8%).

#### 3.3.2. Predictors of success of transition

The results of the univariable analysis indicated that a higher disease activity (as assessed with PGA) and perceived disease burden (as reported by patients) at the time of TSS completion, along with experiencing disease exacerbation after transfer, were associated with lower TSS scores (Table 5). Additionally, patients categorized in the “*Backseat*” group (*n* = 7, 5.3%; mean 20.86) and “*Worried & Insecure*” group (*n* = 6, 4.5%; mean: 22.83) demonstrated significantly (*p* = <0.001) lower TSS scores compared to patients in the “*Conscious & Compliant*” group (*n* = 106, 80.3%; mean 25.45). No significant relationships were found between TSS scores and surgery, time between transfer and first adult care appointment, country of birth, divorced caregivers, independent living, or transferring to an external hospital.

**Table 5:** Patient demographics in relation with total score TSS.

	Inclusion patients	Relation with the TSS		
	*N* (%) or mean (SD)	TSS mean (SD)	Pearson correlation	*p*-value
Sex		*p* = 0.43		
Male, %	78 (49)	Male; 24.9 (2.2)		
		Female; 25.2 SD 1.9		
IBD diagnosis		*p* = 0.91		
Crohn’s disease	89 (55.6)	25.0 SD. 2.0		
Ulcerative colitis	66 (41.3)	25.1 SD. 2.1		
IBD-U	5 (3.1)	24.8 SD. 1.8		
Age at filling in TSS (years)	Mean 19.1 (0.3)		−0.076	0.35
	IQR 0.42			
Age at diagnosis (years)	Mean 14.0 (3.2)		0.008	0.92
	IQR 3.56			
Age at transfer (years)	Mean 18.0 (0.3)		−0.097	0.22
	IQR 0.17			
Educational level (*n* = 150)		*p* = 0.09		
Low (lower secondary)	13 (8.7)	24.31 (2.2)		
Medium (upper secondary)	92 (61.3)	24.93 (2.0)		
High (pre-university)	45 (30)	25.48 (1.7)		
Duration (days) between transfer and completing TSS (*n* = 154)	Mean 369.8 (82.1)		−0.007	0.94
	IOR 106.5			
Disease activity (PGA) at moment of transfer	96 (60)	Remission 25.2 (2.0)		0.268
Remission (PGA; Normal)		Disease activity 24.8 (2.0)		
Disease activity (PGA) at moment of filling in the TSS (*n* = 132)	97 (73.4)	Remission 25.2 (1.8)		**0.02**
Remission (PGA; Normal)		Disease activity 24.2 (2.3)		
Disease exacerbation after transfer (*n* = 132)		Yes; 24.4 (2.0)		**0.02**
Yes	37 (28)	No; 25.3 (1.9)		
Disease burden YA at moment of filling in TSS (*n* = 152)		No complains; 25.7 (1.8)		**<0.001**
Disease burden	97 (63.8)	disease burden; 24.6 (2.1)		
Q-Profile YA by pediatric HCP				0.151
“Conscious & Compliant”	90 (68.2)	25.3 (1.7)		
“Backseat patient”	14 (10.6)	24.2 (2.5)		
“Self-confident & Autonomous”	15 (11.4)	24.5 (2.7)		
“Worried & Insecure”	13 (9.8)	24.6 (1.9)		
Q-Profile YA as scored by adult HCP (*n* = 132)				
“Conscious & Compliant”	106 (80.3)	25.5 (1.5)		
“Backseat patient”	7 (5.3)	20.9 (3.5)		
“Self-confident & Autonomous”	13 (9.8)	24.7 (1.2)		
“Worried & Insecure”	6 (4.5)	22.8 (2.0)		
Family composition (*n* = 152)				
No siblings	8 (5.3)	24.8 (3.0)		
1-2 siblings	117 (77)	25.1 (2.1)		
3-4 siblings	22 (14.5)	24.5 (1.7)		
More than 4 siblings	5 (3.3)	26.0 (0.7)		
Acceptance of disease (*n* = 111)		Yes; 25.2 (1.9)		0.215
Yes, always or often	91 (82)	No; 24.4 (2.7)		

Abbreviations: HCP, healthcare providers; IBD, inflammatory bowel disease; IQR, interquartile range; PGA, Physician Global Assessment; TSS, Transition Success Score; YA, young adult.

Bold values represent significant difference; *p* = <0.05.

## 4. Discussion

In this multicenter validation study conducted in the Netherlands, we developed and validated the TSS, the first instrument for assessing the effectiveness of transition in YA IBD patients. We have demonstrated that the TSS is a reliable and valid tool to measure transition success. YAs who participated in the study achieved high scores, suggesting a high level of successful transition in this population, with a mean score of 25 (range 17-27) and a quarter of the participants even achieving maximal scores (27). Specific patient profiles such as “*Backseat*” and “*Worried & Insecure*” as well as disease activity and burden at the time of TSS completion and exacerbation after transfer, were identified as predictors of unsuccessful transition.

### 4.1. Discussion of the development of the TSS

Our previous Delphi study revealed that stakeholders valued generic outcomes such as self-management over disease-specific characteristics.^16^ Consequently, the TSS was developed as a generic tool, not tied to any specific disease. The TSS items are highly similar to the items identified as relevant in Delphi studies on transition outcomes in other diseases, which identified key items related to independence, adherence, appearance in adult care, and satisfaction.^24,25^ In a patient-based study, 518 YAs with different chronic diseases identified 5 indicators of transition success. These 5 indicators (patient not lost to follow-up, attending scheduled visits in adult care, patient building a trusting relationship with the adult provider, continuing attention for self-management, patient satisfaction with transfer of care) are all included in the TSS in some way.^17^ For diseases such as hemophilia^24,26^ and diabetes,^27^ disease-specific items that monitor disease control such as annual bleeding rate or HbA1c have been suggested to reflect transition success. In IBD, fecal calprotectin is a validated marker of disease activity, but not associated with transition success. A Delphi study on sickle cell disease prioritized general items such as QoL.^28^ Our Delphi group decided to not include QoL in the TSS, but we validated the TSS using QoL as a measure and found a significant correlation between a higher TSS score and better QoL (*r* = 0.242, *p* = 0.004).

Questionnaires have been developed that advocate measuring the success of transition for YAs with diabetes^29^ and another for crossover youth^30^ by measuring their self-management skills across various domains to assess transition success. These questionnaires assume that failure to master self-management skills means that the transition was not successful.^30^ The mastery of pre-transfer skills, such as those measured by the TRAQ, can be compared with these questionnaires.^29^ In consideration of the aforementioned factors, the success of transition measured by the TSS is defined as: The transition to adult care is a success when the YA patient is able to manage their illness independently and knows how to navigate the adult care system with satisfaction. To the best of our knowledge, the TSS is the first instrument that quantitatively measures the success of transition.

### 4.2. Discussion of the validation of the TSS

As is typical of a well-designed questionnaire, the questions from the TSS elicit information at varying levels of knowledge. Questions 1, 8, and 9 provide minimal information, yet the study group, supported by literature,^15,16,19,20,31^ deemed them to be of such relevance, based on the holistic view of transition (satisfaction YA and their caregivers), that they were retained in the TSS. Furthermore, the removal of these questions did not result in significantly altered values in the validation analyses conducted with Cronbach’s alpha and Pearson’s correlations. We demonstrated that the TSS effectively identifies YAs who did not have a successful transition (SD −4 to 0; Rasch range −4 to 4). This is of major importance for routine clinical use, as it can identify at-risk patients who require heightened guidance and care, likely benefitting from a tailored transition program or intervention. Correlation analyses provided support for all hypotheses regarding construct validation, although correlations were predominantly low or moderate, suggesting that the success of transition is influenced by multiple factors. Consistent with prior research findings, a holistic view of the transition period is crucial, with care strategies tailored to individual patients^1–3,9,31–33^ Conversely, it can be argued that QoL, self-management, and knowledge are influenced not only by the manner in which the transition occurs, but also by factors such as disease course and activity and specific patient characteristics, for example, gender and age.^5,6,21^A notable distinction between pediatric and adult healthcare lies in the shift of responsibility from caregivers to the patient in the management of their disease and treatment regimen. As such, the development of self-management skills such as taking medication is important during a transition program.^2,9,32^ Notably, the correlation between the TSS and the self-management tool TRAQ-NL emerged as the strongest, underscoring the importance of enhancing self-management skills within structured transition programs.

### 4.3. Discussion of the patient outcomes

Lower TSS scores were found in patients characterized as “*Backseat patients*,” who prefer to be guided and cared for by their caregivers and often show less interest in managing their illness.^22^ Such coping behavior can diminish independence and impact transition success negatively. Additionally, patients experiencing greater disease burden and more disease exacerbations after transfer exhibited significantly lower TSS scores. This may reflect the consequences of an unsuccessful transition. Or, vice versa, active disease during transfer has been reported to lead to a less successful transition.^15^

### 4.4. Strengths and limitations

A strength of this study is the multicenter prospective design and the robust development of a novel quantitative tool that evaluates the effectiveness of transitional care. All validation steps have been carried out according to the COSMIN methodology with a sufficient number of questionnaires that enable us to draw clear conclusions. Nevertheless, some limitations of this study should be mentioned. Firstly, the absence of a gold standard to measure the success of transition complicates the validation of the TSS and necessitated the use of measurement tools that appear to be scientifically related to the skills needed for successful transition: self-management, knowledge, and QoL. This underscores, the critical need for establishing such a standard to accurately measure success. Secondly, selection bias may apply since all included patients scored high on the TSS. This might be the consequence of the high quality of transitional IBD care in the Netherlands, even though it is not uniform between hospitals. Four hospitals (representing 85% of the included patients) had established multidisciplinary transition clinics or protocols. Alternatively, a considerable proportion of eligible YA (49%) could not be included in the study, with 26% being unable to be contacted and 35% declining to participate (Figure 2). This group of patients differed significantly from those who did participate, primarily comprising patients with less severe disease activity at the time of transfer, often categorized by pediatricians as “*Self-confident & Autonomous*” for whom transition may have been less successful. Therefore, TSS scores from this study might not be representative for the IBD population at large. Further research is required to gain a deeper understanding of the cutoff scores. It is crucial to have a representative sample of the entire IBD patient population, ideally from multiple countries. One potential approach is to obtain consent from patients to participate in the study at an early stage, before the transfer. This way, those patients who will potentially drop out of care later (lost to follow-up), thus having an unsuccessful transition, will still be part of the study sample. Thirdly, VAS scores from both patients and caregivers measuring transition success might be inflated since the survey has been conducted by their own healthcare team. To mitigate this effect, patients and caregivers were asked to fill in the questionnaire online.

## 5. Conclusions

In this study, we developed the first validated quantitative measure of healthcare transition outcomes for YA with IBD. The TSS allows healthcare professionals in adult care to identify YA in whom transfer of care was unsuccessful in order to provide additional tailored transitional care in response. Moreover, the TSS aids in generating risk profiles to pinpoint patients at risk of transition failure and assists in discerning effective interventions or transition programs.

As this TSS is non-disease specific, validation in cohorts of patients with other chronic diseases would be insightful after TSS validation in these disorders as well. We encourage all healthcare providers involved in IBD transitional care to adopt the TSS to monitor in order to evaluate and improve their transition programs.

**Collaborators**

KICC; Patrick F. van Rheenen^1^, Sarah T.A. Teklenburg^2^

ICC; Fiona D.M. van Schaik^3^, Janneke C. van der Woude^4^, Jildou Hoekstra^5^, Marleen de Leest^6^

NIBD; Carla Bakker^4^, Elvira M.E. Besuijen-Laterveer^7^, Esther Adriaanse^5^, Jolien Wisse^6^, Marloes Heida^8^, Pamela Hurkmans^9^, Nynke Boontje^4^, Tessa. Z. Toonen^10^, and Wendy Heida^6^

^1^UMC Groningen – Beatrix Children’s Hospital, Department of Paediatric Gastroenterology, Groningen, The Netherlands; ^2^Isala Hospital, Department of Paediatric, Zwolle, The Netherlands; ^3^UMC Utrecht, Department of Gastroenterology, Utrecht, The Netherlands; ^4^Erasmus MC, Department of Gastroenterology, Rotterdam, The Netherlands; ^5^Amphia Hospital, Department of Gastroenterology, Breda, The Netherlands; ^6^Rijnstate Hospital, Department of Gastroenterology, Arnhem, The Netherlands; ^7^Isala Hospital, Department of Gastroenterology, The Netherlands; ^8^UMC Groningen, Department of Gastroenterology, Groningen, The Netherlands; ^9^Amphia Hospital, Department of Paediatric, Breda, The Netherlands; ^10^UMC Utrecht – Wilhelmina Children’s Hospital, Department of Paediatric Gastroenterology, Utrecht, The Netherlands

## Supplementary Data

Supplementary data are available online at *ECCO-JCC* online.

jjae166_suppl_Supplementary_Table

## Data Availability

The data underlying this article will be shared on reasonable request to the corresponding author.

## Notes

*Conference presentation*: ECCO 2024: poster presentation and oral presentation NECCO. ESPGHAN 2024: oral presentation.

## References

[1] van Rheenen PF , Aloi M, Biron IA, et al European Crohn’s and Colitis Organisation topical review on transitional care in inflammatory bowel disease. J Crohns Colitis 2017;11:1032–8. 28158494 10.1093/ecco-jcc/jjx010

[2] Brooks AJ , Smith PJ, Cohen R, et al UK guideline on transition of adolescent and young persons with chronic digestive diseases from paediatric to adult care. Gut 2017;66:988–1000. 28228488 10.1136/gutjnl-2016-313000 PMC5532456

[3] Philpott JR , Kurowski JA. Challenges in transitional care in inflammatory bowel disease: a review of the current literature in transition readiness and outcomes. Inflamm Bowel Dis 2019;25:45–55. 29893932 10.1093/ibd/izy207

[4] Hait EJ , Barendse RM, Arnold JH, et al Transition of adolescents with inflammatory bowel disease from pediatric to adult care: a survey of adult gastroenterologists. J Pediatr Gastroenterol Nutr 2009;48:61–5. 19172125 10.1097/MPG.0b013e31816d71d8

[5] van Gaalen MAC , van Gijn E, van Pieterson M, de Ridder L, Rizopoulos D, Escher JC. Validation and reference scores of the transition readiness assessment questionnaire in adolescent and young adult IBD patients. J Pediatr Gastroenterol Nutr 2023;77:381–8. 37347146 10.1097/MPG.0000000000003868

[6] van Gaalen MAC , van Pieterson M, van den Brink G, et al Rotterdam transition test: a valid tool for monitoring disease knowledge in adolescents with inflammatory bowel disease. J Pediatr Gastroenterol Nutr 2022;74:60–7. 34371508 10.1097/MPG.0000000000003278

[7] Cole R , Ashok D, Razack A, Azaz A, Sebastian S. Evaluation of outcomes in adolescent inflammatory bowel disease patients following transfer from pediatric to adult health care services: case for transition. J Adolesc Health 2015;57:212–7. 26206442 10.1016/j.jadohealth.2015.04.012

[8] Fishman LN , Ding J. Optimizing the transition and transfer of care in pediatric inflammatory bowel disease. Gastroenterol Clin North Am 2023;52:629–44. 37543405 10.1016/j.gtc.2023.05.004

[9] Kim J , Ye BD. Successful transition from pediatric to adult care in inflammatory bowel disease: what is the key? Pediatr Gastroenterol Hepatol Nutr 2019;22:28–40. 30671371 10.5223/pghn.2019.22.1.28 PMC6333582

[10] Crowley R , Wolfe I, Lock K, McKee M. Improving the transition between paediatric and adult healthcare: a systematic review. Arch Dis Child 2011;96:548–53. 21388969 10.1136/adc.2010.202473

[11] Coyne B , Hallowell SC, Thompson M. Measurable outcomes after transfer from pediatric to adult providers in youth with chronic illness. J Adolesc Health 2017;60:3–16. 27614592 10.1016/j.jadohealth.2016.07.006

[12] Cleverley K , Rowland E, Bennett K, Jeffs L, Gore D. Identifying core components and indicators of successful transitions from child to adult mental health services: a scoping review. Eur Child Adolesc Psychiatry 2020;29:107–21. 30294756 10.1007/s00787-018-1213-1 PMC7024692

[13] Pearlstein H , Bricker J, Michel HK, et al Predicting sub-optimal transitions in adolescents with inflammatory bowel disease. J Pediatr Gastroenterol Nutr 2020;72:563–8. 10.1097/MPG.0000000000003013 33264185

[14] van Staa A , Sattoe JN. Young adults’ experiences and satisfaction with the transfer of care. J Adolesc Health 2014;55:796–803. 25149686 10.1016/j.jadohealth.2014.06.008

[15] van den Brink G , van Gaalen MAC, Zijlstra M, de Ridder L, van der Woude CJ, Escher JC. Self-efficacy did not predict the outcome of the transition to adult care in adolescents with inflammatory bowel disease. Acta Paediatr 2019;108:333–8. 29926962 10.1111/apa.14471 PMC6585705

[16] van den Brink G , van Gaalen MAC, de Ridder L, van der Woude CJ, Escher JC. Health care transition outcomes in inflammatory bowel disease: a multinational Delphi study. J Crohns Colitis 2019;13:1163–72. 30766997 10.1093/ecco-jcc/jjz044 PMC7142327

[17] Sattoe JNT , Hilberink SR, van Staa A. How to define successful transition? An exploration of consensus indicators and outcomes in young adults with chronic conditions. Child Care Health Dev 2017;43:768–73. 28074484 10.1111/cch.12436

[18] Mokkink LB , Terwee CB, Patrick DL, et al The COSMIN checklist for assessing the methodological quality of studies on measurement properties of health status measurement instruments: an international Delphi study. Qual Life Res 2010;19:539–49. 20169472 10.1007/s11136-010-9606-8 PMC2852520

[19] Erős A , Soós A, Hegyi P, et al Spotlight on transition in patients with inflammatory bowel disease: a systematic review. Inflamm Bowel Dis 2020;26:331–46. 31504524 10.1093/ibd/izz173

[20] Sattoe JNT , Peeters MAC, Haitsma J, van Staa AL, Wolters VM, Escher JC. Value of an outpatient transition clinic for young people with inflammatory bowel disease: a mixed-methods evaluation. BMJ Open 2020;10:e033535. 10.1136/bmjopen-2019-033535 PMC6955474 31911522

[21] Russel MG , Pastoor CJ, Brandon S, et al Validation of the Dutch translation of the inflammatory bowel disease questionnaire (IBDQ): a health-related quality of life questionnaire in inflammatory bowel disease. Digestion 1997;58:282–8. 9243124 10.1159/000201455

[22] Jedeloo S , van Staa A, Latour JM, van Exel NJ. Preferences for health care and self-management among Dutch adolescents with chronic conditions: a Q-methodological investigation. Int J Nurs Stud 2010;47:593–603. 19900675 10.1016/j.ijnurstu.2009.10.006

[23] Baker F. The Basics of Item Response Therapy. University of Maryland, College Park: ERIC Clearinghouse on Assessment and Evaluation; 2001.

[24] Suris JC , Akre C. Key elements for, and indicators of, a successful transition: an international Delphi study. J Adolesc Health 2015;56:612–8. 26003575 10.1016/j.jadohealth.2015.02.007

[25] Fair C , Cuttance J, Sharma N, et al; International and Interdisciplinary Health Care Transition Research Consortium. International and interdisciplinary identification of health care transition outcomes. JAMA Pediatr 2016;170:205–11. 26619178 10.1001/jamapediatrics.2015.3168 PMC6345570

[26] Sun HL , Breakey VR, Straatman L, Wu JK, Jackson S. Outcomes indicators and processes in transitional care in adolescents with haemophilia: a Delphi survey of Canadian haemophilia care providers. Haemophilia 2019;25:296–305. 30817086 10.1111/hae.13699

[27] Pierce J , Aroian K, Schifano E, Gannon A, Wysocki T. Development and content validation of the healthcare transition outcomes inventory for young adults with type 1 diabetes. J Patient Rep Outcomes 2019;3:71. 31858284 10.1186/s41687-019-0163-9 PMC6923308

[28] Sobota AE , Shah N, Mack JW. Development of quality indicators for transition from pediatric to adult care in sickle cell disease: a modified Delphi survey of adult providers. Pediatr Blood Cancer 2017;64:6. 10.1002/pbc.26374 27905689

[29] Pierce J , Hossain J, Gannon A. Validation of the healthcare transition outcomes inventory for young adults with type 1 diabetes. J Pediatr Psychol 2020;45:767–79. 32642778 10.1093/jpepsy/jsaa051

[30] Dubov V , Agnihotri S, Goodman D, Patel M. Development of the Successful Transitions Assessment Tool (STAT) for Crossover Youth (COY): youth voice and expert feedback with a Delphi approach. Child Abuse Negl 2022;134:105900. 36179381 10.1016/j.chiabu.2022.105900

[31] Bihari A , Olayinka L, Kroeker KI. Outcomes in patients with inflammatory bowel disease transitioning from pediatric to adult care: a scoping review. J Pediatr Gastroenterol Nutr 2022;75:423–30. 35920854 10.1097/MPG.0000000000003581 PMC9470050

[32] Goodhand J , Hedin CR, Croft NM, Lindsay JO. Adolescents with IBD: the importance of structured transition care. J Crohns Colitis 2011;5:509–19. 22115368 10.1016/j.crohns.2011.03.015

[33] Paine CW , Stollon NB, Lucas MS, et al Barriers and facilitators to successful transition from pediatric to adult inflammatory bowel disease care from the perspectives of providers. Inflamm Bowel Dis 2014;20:2083–91. 25137417 10.1097/MIB.0000000000000136 PMC4328150

