# Supplementary material for: Effectiveness of Transitional Care in Inflammatory Bowel Disease; Development, Validation, and Initial Outcomes of a Transition Success Score
Source: J Crohns Colitis. 2024 Nov 1;19(4):jjae166. doi: 10.1093/ecco-jcc/jjae166 (PMC12041418; doi:10.1093/ecco-jcc/jjae166)
Supplement: jjae166_suppl_Supplementary_Table [file jjae166_suppl_supplementary_table.docx]

Supplemental table 1; Demographic characteristics of the expert panel (n=60)

|  |  | N (%) |
| --- | --- | --- |
| Sex | Male, % | 24 (40) |
| Hospital type | Non-academic hospital  Academic hospital | 5 (8.3)  55 (91.7) |
| Department | Paediatrics  Gastroenterology  Internal Medicine | 36 (60)  18 (30)  6 (10 |
| Position | Paediatric Gastroenterologist  Paediatric IBD Nurse  Gastroenterologist  Adult IBD Nurse  Clinical research fellow  Transition manager | 25 (41.7)  9 (15)  19 (31.7)  5 (8.3)  1 (1.7)  1 (1.7) |
| Continent | Europe  North America  Asia | 48 (80)  6 (10)  6 (10) |
